# Modulation of Motor Area Activity by the Outcome for a Player during Observation of a Baseball Game

**DOI:** 10.1371/journal.pone.0008034

**Published:** 2009-11-25

**Authors:** Sotaro Shimada

**Affiliations:** Department of Electronics and Bioinformatics, School of Science and Technology, Meiji University, Kawasaki, Kanagawa, Japan; The University of Western Ontario, Canada

## Abstract

**Background:**

Observing competitive games such as sports is a pervasive entertainment among humans. The inclination to watch others play may be based on our social-cognitive ability to understand the internal states of others. The mirror neuron system, which is activated when a subject observes the actions of others, as well as when they perform the same action themselves, seems to play a crucial role in this process. Our previous study showed that activity of the mirror neuron system was modulated by the outcome of the subject's favored player during observation of a simple competitive game (rock-paper-scissors). However, whether the mirror neuron system responds similarly in a more complex and naturalistic sports game has not yet been fully investigated.

**Methodology/Principal Findings:**

In the present study, we measured the activity of motor areas when the subjects, who were amateur baseball field players (non-pitchers), watched short movie clips of scenes in professional baseball games. The subjects were instructed to support either a batter or a pitcher when observing the movie clip. The results showed that activity in the motor area exhibited a strong interaction between the subject's supported side (batter or pitcher) and the outcome (a hit or an out). When the subject supported the batter, motor area activity was significantly higher when the batter made an out than when he made a hit. However, such modulation was not apparent when the subject supported the pitcher.

**Conclusions/Significance:**

This result indicates that mirror neuron system activity is modulated by the outcome for a particular player in a competitive game even when observing a complex and naturalistic sports game. We suggest that our inclination to watch competitive games is facilitated by this characteristic of the mirror neuron system.

## Introduction

Most of us enjoy watching competitive games performed by others, such as sports, car races or chess, as well as playing them ourselves. For example, we may occasionally go to a stadium to watch professional sports, such as football, baseball, or boxing. There are also famous worldwide competitions, such as the Olympics or the soccer World Cup, which are enthusiastically watched by audiences all over the world. Thus, there is no doubt that watching competitive games is a pervasive entertainment among humans. Nevertheless, the reason we like watching competitive games is not clear.

Our inclination to watch competitive games most likely relies, at least in part, on our social-cognitive ability to understand or share the internal motor and sensory states of others [1], [2]. This process is thought to be realized by the brain areas collectively known as the mirror neuron system (MNS), which is activated not only when an observer performs motor actions themselves, but also when they watch the same actions performed by others [3]. Mirror neurons were first found in the monkey F5 area in premotor cortex, and subsequent studies confirmed homologous human activity in the premotor, parietal, and primary motor areas [4]–[7]. By internally duplicating, for example, a sports player's motor representation, observers can vicariously experience a player's internal state as if they were playing in the game themselves. We thus consider that the MNS is one of the most relevant neural mechanisms active during the observation of competitive games. One intriguing question concerning the observation of competitive games is whether MNS activity is modulated by the status of the observer, that is, the observer's preference for a particular player. Note that this problem is critical in the multi-person action observation situation but absent in the single-person situation that most previous MNS studies have addressed.

Our previous study showed that MNS activity during observation of a competitive game was modulated by the outcome for the player who the subject supported [8]. In this previous experiment, the subject watched a movie clip of a simple competitive game (Rock-Paper-Scissors, RPS) performed by two players. The subject was instructed to watch the movie as if they were supporting one player with a concordant view from the subject. Results showed that the motor area (considered a part of the MNS) was activated more when the subject's supported player won than when this player lost. This result indicates that MNS activity was not uniform during observation of competitive games performed by multiple players but was modulated by the outcome for the subject-supported player in the game. However, because the previous study employed a relatively simple competitive game and a laboratory experimental (non-naturalistic) setting to film the movie stimuli, it was unclear whether similar MNS activity is observed for more naturalistic and complex competitive games like professional sports game. Indeed, RPS is also naturalistic because it is played in daily life situations. However, we suggest that professional sports games need to be investigated specifically because they are often viewed by large audiences, either at a stadium or on television. Examination of brain activity during viewing such naturalistic visual stimuli is important for understanding human cognition [9]. From a technical viewpoint, a professional sports game usually includes whole-body complex motion and interaction among other players, which is not apparent in the RPS game. It has been reported that whole-body complex motion (i.e., dance) also activates the MNS [10]–[12]. However, whether this activity is modulated by interaction among multiple models has not been investigated.

The purpose of this study was to examine MNS activity during observation of a naturalistic competitive game. MNS activity during observation of a professional baseball games was measured using near-infrared spectroscopy (NIRS). Baseball was chosen as the stimuli in our experiment because it is popular and frequently broadcasted in the author's country (Japan) so that naturalistic stimuli (i.e., from Television) were easily obtained. We recruited amateur baseball players as subjects because MNS activity is known to be affected by the subjects' experience of the observed action [10]–[12], and we wanted to maximize the MNS activity during observation and to minimize the variance of this effect in our experiment. All subjects were field players (non-pitcher) and were instructed to observe the movie clip as if they were supporting the batter to see whether MNS activity was modulated by the outcome for this player. We also examined MNS activity when the subject supported the pitcher. We predicted that MNS activity would be modulated by the outcome for a particular player even in a more naturalistic competitive game as baseball, and more specifically, that MNS activity would be higher when the subject-supported batter hits than when this batter makes an out, similarly to our previous study.

## Materials and Methods

### Participants

Twelve healthy male subjects participated in the experiment (aged 20.5±2.5 years; mean±SD). All subjects were members of an amateur baseball team and had played baseball for more than 3 years (7–8 years for most of them). All subjects were field players (not a pitcher) and practiced baseball about twice a week at the time of the experiment. All but one subject were right-handed, and they had normal or corrected-to-normal vision. Additional two male subjects who had the experience of practicing baseball for more than 3 years participated in the control experiment. Written informed consent was obtained from all subjects. The experiments were approved by the ethics committee of the School of Science and Technology, Meiji University, and conducted according to the principles and guidelines of the Declaration of Helsinki.

### Stimuli

Stimuli were collected from televised Japanese professional baseball games. Scenes in which a right-handed pitcher has a right-handed batter as an opponent were collected. We selected 10 scenes, in which the batter made a grounder hit (HIT condition), and 10 scenes, in which the batter made a ground out (OUT condition). The movie clip included only the sequence of scenes where (1) the pitcher threw a ball and (2) the batter hit the ball, but it did not include scenes where a fielder caught the ball. Therefore, the stimuli were highly similar between HIT and OUT conditions. Although the subjects were not informed explicitly of the outcome of the match-up, the subjects could recognize whether the outcome was a hit or an out because the subjects had sufficient experience with baseball, and relatively salient scenes (easy to infer the result by seeing the initial trajectory of the ball) were selected. The length of each movie clip was 4 sec and included no cuts (changes of camera angle). The camera angle was fixed throughout the scene, and the scene consisted of the backside view of the pitcher and the batter facing toward the pitcher. After the main experiment, the subjects were asked to judge whether the outcome of the match-up was a hit or an out in a forced-choice manner by observing the same stimuli used in the main experiment. The correct response rate was 94.1±7.0% (mean±SD), indicating that the subjects were highly accurate in judging the outcome by observing only the hitting scene (without the outcome scene).

### Procedure

The experiment was performed in a quiet room. The movie clips were displayed on a liquid-crystal color monitor (TL32WRJ-B, Uniden, Japan). Subjects were seated comfortably on a chair in front of the monitor. The viewing distance was approximately 2 m. Each movie clip lasted 4 s, and the interstimulus interval was 10 s, during which a blank screen was presented (rest period). The experimental session consisted of 20 trials: 10 trials each for the two conditions (HIT or OUT). In the first session, the subjects were instructed to support the batter (BATTER session), and in the second session, the subjects were instructed to support the pitcher (PITCHER session). The same movie clips were used in the two sessions, although the order of the presentation was pseudorandom in both sessions. To summarize, there were 4 conditions in our experiment: BATTER-HIT, BATTER-OUT, PITCHER-HIT, and PITCHER-OUT (support-outcome pair), where BATTER-HIT represents the condition in which the subject supported the batter and the outcome was a hit. Because all subjects were more specialized as a batter (not a pitcher), our main interest was examining brain activity during the BATTER session; thus the subject underwent the BATTER session first. However, to examine whether the order of sessions contaminated the brain responses, we also conducted a control experiment that was the same as the main experiment, except that the order of sessions was reversed.

### NIRS Recordings

**Figure 1:**
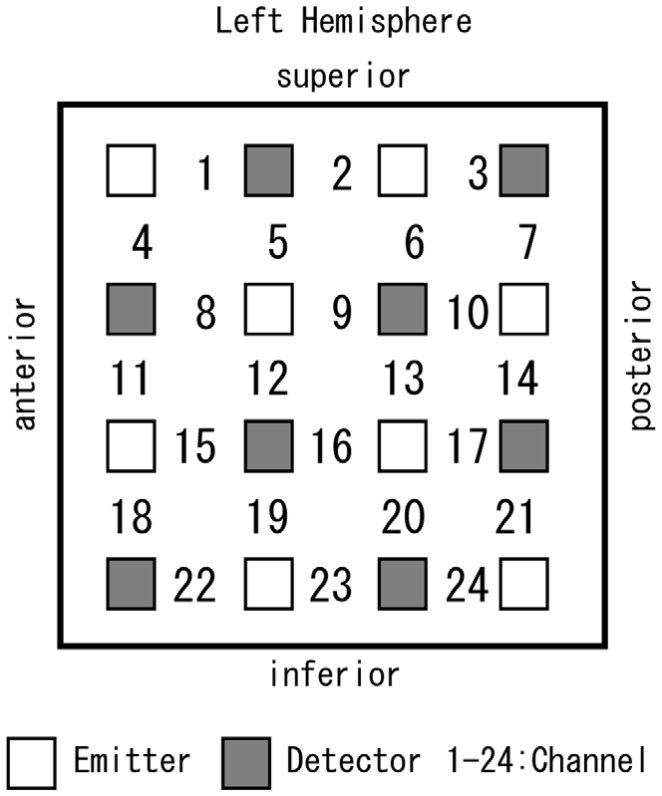
Location of the NIRS optodes placed on the motor areas in the left hemisphere. The distance between each emitter and the corresponding detector was set at 3 cm. C3 was located at the center between channels 9 and 10.

NIRS measurements were performed throughout the experiment. A multichannel NIRS unit operating at 780, 805, and 830 nm wavelengths (OMM-3000, Shimadzu, Kyoto, Japan) was used to measure temporal changes in concentrations of oxyhemoglobin (oxy-Hb), deoxyhemoglobin (deoxy-Hb), and total hemoglobin (total-Hb). Sixteen optodes constituted 24 channels and were placed on the motor area of the left hemisphere, including C3 of the international 10/20 system at the center (9×9 cm square area, Fig. 1). Each channel consisted of one emitter optode and one detector optode located 3 cm apart from the emitter. The sampling rate at each channel was approximately 8 Hz. These channels were likely placed on the pre- or postcentral gyrus [13], and in this paper, the measured area is simply referred to as the motor area.

### Statistical Analysis

Statistical analyses of the NIRS data were performed with a least squares estimation using a general linear model (GLM) [7], [14] implemented with Matlab 7.5 (MathWorks, Natick, MA). The design matrix employed one 2-s–delayed box-car function for each experimental condition, convolved with a Gaussian kernel of dispersion of 4-s full-width half-maximum, which modeled temporal correlations in an NIRS time series. The AR(1) model was used to adjust for autocorrelated error terms. The experimental condition period (action observation) was contrasted against the rest period. The resulting contrasts (t-values) for each condition were submitted to 2 (outcome)×2 (support) repeated-measures analyses of variance (ANOVA) to see whether there was a main effect of outcome of the match-up and/or subjective support for a player or an interaction between these 2 factors. All statistical threshold levels were set at P<0.05, with a false discovery rate (FDR) control for multiple-comparison adjustments [15]. Although each NIRS parameter was analyzed, we mainly report here the results of oxy-Hb because we consider oxy-Hb to be the most sensitive parameter of hemodynamic responses [16], [17].

## Results

**Figure 2:**
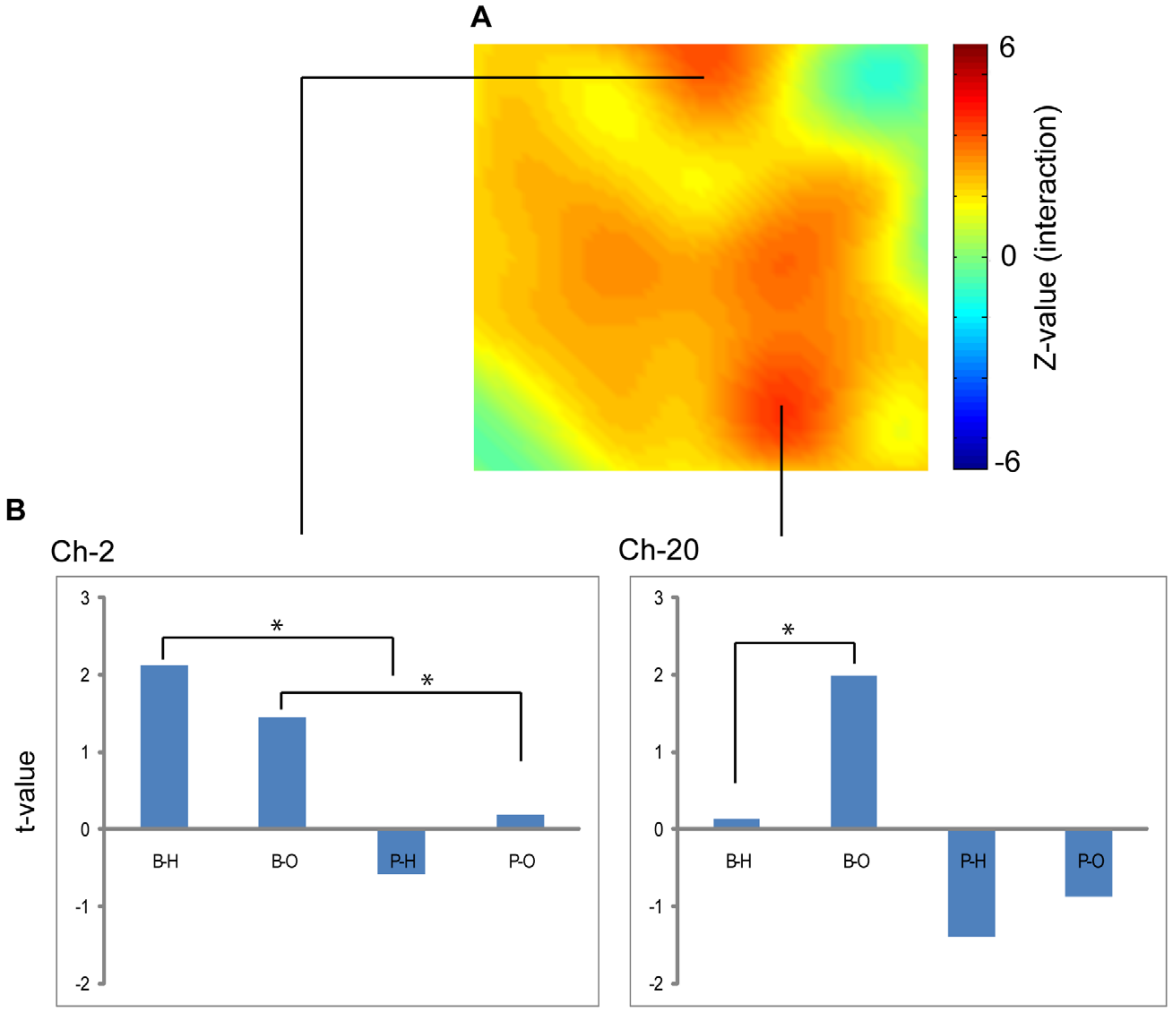
Results of NIRS measurement. A. Z-map (oxy-Hb) of channels that showed an interaction between appearance and kinematics factors thresholded at P<0.05 (corrected). B. Z-values for each condition contrasted against the control period calculated by GLM analyses at ch-2 and ch-20. B-H, Batter-Hit; B-O, Batter-Out; P-H, Pitcher-Hit; P-O, Pitcher-Out conditions.

There was no significant main effect of support side or outcome, but there were significant interactions between these factors in motor area activity (P<0.05, corrected; Fig. 2; Table 1). Subsequent post-hoc analyses in ch-20, which showed the highest interaction (F_(1, 11)_ = 76.5, P<0.001, corrected), revealed that there was a significant difference between the HIT and OUT conditions in the BATTER session (F_(1, 22)_ = 7.93, P = 0.01) but not in the PITCHER session (F_(1, 22)_ = 0.70, P = 0.41). Random effect analyses revealed that there was a significant activation in the BATTER-HIT condition (t_(11)_ = 1.99, p = 0.04) but not in the other three conditions (P>0.1). A similar activation pattern was observed in the adjacent channels (ch-13 and ch-17; Table 1).

**Table 1:** Statistical results of the 2×2 analysis of variance for each NIRS parameter.

		Outcome		Support		Interaction (outcome × support)	
		F(1, 11)	P*uncorr*	F(1, 11)	P*uncorr*	F(1, 11)	P*uncorr*
ch-2	oxyHb	5.75	0.04	9.52	0.01	57.6	<0.001*
	deoxyHb	5.72	0.04	18.7	0.001*	15.9	0.002*
	totalHb	4.06	0.07	4.84	0.05	25.4	<0.001*
ch-12	oxyHb	4.20	0.07	2.08	0.2	24.0	<0.001*
	deoxyHb	1.37	0.3	0.25	0.6	1.45	0.3
	totalHb	6.67	0.03	0.74	0.4	30.2	<0.001*
ch-13	oxyHb	7.49	0.02	1.40	0.3	38.5	<0.001*
	deoxyHb	2.43	0.15	2.03	0.18	11.2	0.007
	totalHb	6.53	0.03	1.12	0.3	57.7	<0.001*
ch-17	oxyHb	4.19	0.07	4.54	0.06	19.3	0.001*
	deoxyHb	1.81	0.2	9.39	0.01	2.5	0.14
	totalHb	1.34	0.3	0.52	0.5	10.0	0.009
ch-20	oxyHb	9.44	0.01	1.83	0.2	76.5	<0.001*
	deoxyHb	2.16	0.17	1.82	0.2	17.7	0.001*
	totalHb	7.17	0.02	1.30	0.3	111.1	<0.001*

P*uncorr*: uncorrected P-value.

*P<0.05, corrected with false discovery rate (FDR) control.

Another activation focus that showed the interaction was located on ch-2 (F_(1, 11)_ = 57.6, P<0.001, corrected). There was a significant activation in BATTER-HIT condition (t_(11)_ = 2.13, P = 0.03) and a marginal activation in BATTER-OUT condition (t_(11)_ = 1.45, P = 0.09) but not in PITCHER conditions (P>0.1). There were significant differences between BATTER-HIT and PITCHER-HIT conditions (F_(1, 22)_ = 13.5, P = 0.002) and between BATTER-OUT and PITCHER-OUT conditions (F_(1, 22)_ = 5.64, p = 0.03). However, there were no significant simple main effects of outcome (HIT/OUT), both in the BATTER and PITCHER sessions (P>0.1).

The additional two subjects, who participated in the PITCHER session first (control experiment), showed similar brain responses. In ch-2 of both subjects, the t-value for each condition was not different from the 12 subjects who participated in the main experiment (P>0.05, two-tailed t-test). In ch-20, t-values were also not different from those in the main experiment, except that one subject in the control experiment showed a higher activity in the PITCHER-HIT condition (t_(11)_ = 2.54, P = 0.03) although GLM analysis showed that it did not differ from the activity in the PITCHER-OUT condition of this subject (t_(2281)_ = 1.12, P>0.1). This suggests that the order of the sessions have little effect on brain responses in the current experiment.

## Discussion

The results showed that the subject's supported side and the outcome of the match-up modulated the motor area activity. The motor area showed a significant difference between HIT and OUT conditions in the batter-supported session, but this difference was not apparent in the pitcher-supported session. Because visual features of the stimuli were highly similar between conditions, it is unlikely that this differential activation was caused by early visual processing. Rather, the brain activity likely reflected the ‘resonance’ activity of the MNS caused by the action of the subject's supported player because the activation foci was located in the motor area near C3 of the 10/20 system. In the present experiment, however, we did not examine brain activity when the subjects themselves performed the same action because of the technical difficulty in measuring brain activity without motion artifacts when swinging a bat. Nevertheless, because the significant activation was found in brain areas (slightly posterior to C3) similar to the previous study [8], we believe that the observed brain activity reflected the activity in the motor area comprising the MNS. It is also consistent with several previous studies that reported the MNS property in the motor area near C3 [4]–[8].

While the motor area was activated when the subjects supported the batter, no such activation was observed during the pitcher-supported session. There could be several reasons for this result. First, the subjects were field players, who were more specialized in hitting than in pitching, and so the subject's MNS was more sensitive to the outcome for hitting actions than for pitching actions. It is known that MNS activity is affected by the subject's expertise on the observed action [10], [11], and our result seems consistent with these studies. Second, the orientation of the body of the player may affect MNS activity. Kilner et al. [18] showed that MNS activity in action observation was higher when the actor faced towards the observer than when the actor showed their back to the observer. In the stimuli of our experiment, the pitcher showed their back while the batter faced towards the observer. Therefore, it is possible that MNS activity was not sensitive to the outcome for the player when observing actions of the pitcher compared with those of the batter. However, since the scene in which the pitcher faces towards the camera (and the batter shows their back) or a lateral view of the players is rarely broadcasted, we could not further examine this effect in the current experiment. Finally, the pitcher-supported session followed after the batter-supported session in our experiment, so the motor area activity may be affected by session-order effects. However, because ANOVA showed that there was no main effect of supported side (BATTER or PITCHER), we believe that overall brain activity was not affected by the order of sessions. In addition, the control experiment showed that the brain response was similar when the order of sessions was reversed.

Although the present result was consistent with previous findings in that the motor area activity was modulated by the outcome of a competitive game, the activity pattern in the motor area was somewhat different from the previous study [8]. In the previous study, the motor area activity was higher when the subject's supported player won than when he lost. In the present study, ch-2 showed a similar activity with the significant activation in BATTER-HIT condition, although we failed to show a significant difference between BATTER-HIT and BATTER-OUT conditions. Contrary, ch-20 and its adjacent channels (ch-13 and 17) were more activated when the supported player lost (OUT condition) than when he won (HIT condition). The difference in experimental settings between the present and previous studies may cause this different activity pattern in the motor area. There were at least three differences in experimental settings between the two studies: First, in the previous study, the stimuli were recorded in a laboratory setting; the stimuli included only the right forearm and hand of two players, which made a hand gesture (rock, paper, or scissors) in a well-controlled way. In contrast, the stimuli in the present study were extracted from televised professional baseball games, in which the entire bodies of the batter and the pitcher were presented, performing proficient and complicated actions. This could enable the skilled observer to attend to the subtle difference in the player's movement, which may be reflected in the MNS activity. Second, the outcome (win or lose) was immediately observable in the RPS game, while the outcome was not directly presented, but had to be inferred, in the baseball stimuli in the current experiment because the outcome scene was not presented. Although the outcome was easily inferred by the subjects (94%), this inference should be based on the internal simulation of the trajectory of the ball, which likely involves activation of the MNS as well as other brain areas. Third, the present subjects were experts (compared with normal subjects) in performing the action observed (hitting), while the subjects in the previous study were not experts in the RPS game. Interestingly, some subjects reported that they noticed an ill-balanced movement of the batter in OUT conditions in the informal interview after the experiment. Taken together, the present experimental settings were more naturalistic than the previous one, in that the observer who is highly familiar and experienced with the sport watched a naturalistic scene of a professional game.

While several previous studies reported that MNS activity was higher in correct/desirable response conditions [8], [19], some other studies showed a reversed activity pattern (higher in erroneous responses) [20]. In this sense, the present result was concordant with the second group of studies, that is, the motor area (ch-20 and adjacent areas) activity was higher when observing erroneous actions (the batter made an out). We speculate that higher activation during observation of correct actions reflects the tendency to vicariously experience the winner's action, while higher activation during observation of erroneous actions may be caused by brain activity to bridge a gap between observed and desirable actions. We often learn from observing other's erroneous action. One developmental study seems to support our speculation: observation of an erroneous action is sufficient to enact the correct action even in eighteen-month-old children [21]. Similarly, higher motor area activation was reported when observing nonpracticed guitar chords than practiced ones in an imitation-learning paradigm [22]. These findings suggest that the MNS does not simply internally duplicate the model's performance but more actively engages in processing (understanding, imitating, learning, empathizing, etc.) of the observed action [3], [10], [18], [19], [22]. Further study is obviously needed to examine what factors are important for modulating MNS involvement in action observation.
